# The Greek Version of the Mild Behavioral Impairment Checklist (MBI-C): Psychometric Properties in Mild Cognitive Impairment Due to Alzheimer’s Disease

**DOI:** 10.3390/brainsci15050462

**Published:** 2025-04-27

**Authors:** Efthalia Angelopoulou, Evangelia Stanitsa, Maria Hatzopoulou, Akylina Despoti, Niki Tsinia, Vasiliki Kamtsadeli, Marina Papadogiani, Vasilis Kyriakidis, Sokratis Papageorgiou, John D. Papatriantafyllou

**Affiliations:** 1 1st Department of Neurology, Aiginition University Hospital, Medical School, National and Kapodistrian University of Athens, 11528 Athens, Greece; angelthal@med.uoa.gr (E.A.); eva.st.92@gmail.com (E.S.); sokpapa@med.uoa.gr (S.P.); 2 Third Age Day Care Center IASIS, 16562 Athens, Greece; mariahatzopoulou@yahoo.gr (M.H.); a.despoti@yahoo.com (A.D.); ntsinia@gmail.com (N.T.); vkamtsadeli@gmail.com (V.K.); mpapadogianni@outlook.com (M.P.); billkyriakidis5@gmail.com (V.K.); 3 1st Department of Psychiatry, Aiginition University Hospital, Medical School, National and Kapodistrian University of Athens, 11528 Athens, Greece

**Keywords:** mild behavioral impairment, Alzheimer’s disease, mild cognitive impairment, dementia, cognitive decline, mild behavioral impairment checklist

## Abstract

Background/Objectives: Mild behavioral impairment (MBI) is an early marker of Alzheimer’s disease (AD) and other neurodegenerative diseases, often preceding cognitive decline. The MBI Checklist (MBI-C) is a 34-item tool designed to detect MBI. This study aimed to assess the psychometric properties of the Greek version of the MBI-C and its ability to differentiate patients with mild cognitive impairment due to AD (MCI-AD) from cognitively unimpaired older adults (healthy participants, HPs). Methods: A total of 181 participants (104 MCI-AD, 77 HPs) were recruited from the Third Age Day Care Center IASIS (2019–2023), accompanied by a close informant. Participants underwent neuropsychological assessment [Mini-Mental State Examination (MMSE), Addenbrooke’s Cognitive Examination-Revised (ACE-R)], and informants completed the MBI-C. Internal consistency was evaluated using Cronbach’s α and known-group validity was assessed via comparing MBI-C between the MCI-AD and HPs groups. Diagnostic accuracy was determined via receiver operating characteristic (ROC) analysis. Results: The Greek MBI-C showed excellent internal consistency (Cronbach’s α = 0.899). Among its domains, impulse dyscontrol demonstrated the highest reliability (α = 0.901), whereas decreased motivation (α = 0.564) and abnormal perception/thought content (α = 0.617) exhibited lower reliability. MBI-C total and domain scores were significantly higher in patients with MCI-AD than HPs (*p* < 0.001). The area under the curve (AUC) was 0.871 (optimal cutoff = 9.5), indicating excellent diagnostic performance. Conclusions: Overall, the Greek MBI-C has strong psychometric properties for MCI-AD. Sociocultural factors might influence symptom identification and reporting, particularly in the domains of decreased motivation and abnormal perception/thought content. Future research should investigate its predictive value for dementia conversion and its applicability to other populations, including individuals with subjective cognitive decline and non-AD causes of MCI.

## 1. Introduction

Neuropsychiatric symptoms (NPSs), such as irritability, anxiety, depressive mood, apathy, agitation, and delusions, are highly prevalent in those with Alzheimer’s disease (AD) and other dementias, affecting up to 97% of cases during the disease course [1]. Traditionally, NPSs have been considered an intrinsic feature of dementia, being associated with increased morbidity, mortality, institutionalization, and caregiver burden [1]. However, less attention has been given to NPSs in prodromal stages.

Emerging evidence suggests that NPSs may precede the onset of cognitive decline in patients with AD and other dementias [2]. In particular, depression is a well-established risk factor for dementia; however, it remains unclear whether it represents an intrinsic prodromal stage or contributes to the development of neurodegenerative diseases leading to cognitive decline [3]. The proposed underlying mechanisms include hippocampal damage due to glucocorticoids, impaired neurogenesis and synaptic plasticity, chronic inflammation, vascular damage and neurotransmitter imbalance. Additionally, lifestyle factors, such as physical inactivity, social isolation, and poor diet may further exacerbate both conditions [4]. Approximately 28% of patients with a neurodegenerative disease initially receive a psychiatric diagnosis, with depression being the most common condition [5]. As expected, the behavioral variant of frontotemporal dementia (bvFTD) displays the highest misclassification rate (around 50%), followed by semantic dementia (24%) and AD (23%) [5]. Interestingly, a study in Greece found that patients with amnestic or amnestic plus mild cognitive impairment (MCI), compared to their pre-existing personality traits, exhibited increased neuroticism and decreased extraversion and conscientiousness, a pattern similar to that seen in AD dementia [6]. Conversely, patients with bvFTD showed decreased neuroticism, suggesting that personality changes might be inherently linked to disease-specific neurodegenerative processes [6].

According to the National Institute on Aging and Alzheimer’s Association (NIA-AA) Research Framework, individuals in Stage 2 of AD primarily exhibit mild behavioral changes rather than noticeable cognitive decline, as outlined in the 2018 [7] and the updated 2024 criteria [8]. MCI progresses to dementia at an estimated annual rate of 10–15%, with this risk increasing to 25% in individuals with coexisting NPSs [9]. In this context, the emergence of persistent NPSs in later life may indicate an at-risk state for dementia, emphasizing the importance of early behavioral assessment in clinical practice [9].

The International Society to Advance Alzheimer’s Research and Treatment—AA (ISTAART-AA) defines mild behavioral impairment (MBI) as the onset of NPSs in adults aged 50 years or older, representing a clear departure from their usual personality or behavior and persisting for at least six months [10]. These changes fall into five different domains: (1) decreased motivation (indifference, apathy, aspontaneity), (2) emotional dysregulation (anxiety, euphoria, dysphoria, changeability), (3) impulse dyscontrol (disinhibition, agitation, obsessiveness, gambling, stimulus bind behavior, behavioral perseveration), (4) social inappropriateness (loss of empathy and insight, exaggeration of previous personality traits, rigidity), and (5) abnormal perception or thought content (hallucinations, delusions). For an MBI diagnosis, the individual must not have dementia, while MBI can coexist with subjective cognitive decline (SCD) or MCI. Additionally, these behavioral changes must not be attributable to another medical condition, medications, substance abuse, or psychiatric disorder (e.g., major depression, generalized anxiety disorder, schizophrenia) [10].

Various scales have been used to assess MBI, including the interview-based Neuropsychiatric Inventory (NPI), the informant-based NPI Questionnaire (NPI-Q), and the Behavioral Pathology in Alzheimer’s Disease (BEHAVE-AD) [10]. However, these tools were designed for patients with dementia and utilize shorter reference periods (less than six months), increasing the likelihood that NPSs are linked to transient stressful life events rather than persistent behavioral changes [10]. The Mild Behavioral Impairment Checklist (MBI-C) is the first tool specifically designed to assess MBI in functionally independent older adults based on the ISTAART-AA criteria [10]. It consists of 34 items, corresponding to the five MBI domains, and can be completed by a close informant (primarily), the patient, or a clinician. Each item requires a “yes” or “no” response, followed by a severity rating (1 = mild, 2 = moderate, 3 = severe). A “yes” response is selected only if the behavior represents an impactful change from the individual’s long-standing usual behavior and has persisted for at least six months, either continuously or intermittently. The MBI-C can be used in both clinical and research settings to detect and monitor newly emerging NPSs in populations prior to dementia, potentially identifying individuals at increased risk for cognitive decline [10].

Since no prior instrument was specifically designed for MBI assessment, there is no established benchmark for comparison with the MBI-C [10]. The MBI-C has been validated in populations with MCI [11] and SCD [12], with cutoff scores of 6.5 and 8.5 points, respectively, for defining MBI. Additionally, its five-factor structure has been validated in cognitively unimpaired older individuals, while cutoff scores for this population remain uncertain [13].

As the definition and criteria for MBI have been developed relatively recently, the MBI-C has primarily been validated and used in English-speaking populations. However, cultural and linguistic factors, including ethnicity, religion, social norms, traditions, community health behaviors, perceptions of health and illness, coping strategies, and diverse help-seeking behaviors may affect the recognition, reporting, perceived severity, and potential clinical significance of behavioral symptoms [14]. Hence, to fully understand the epidemiology, characteristics, genetic background, pathophysiology, and role of MBI in cognitive decline, studies examining the applicability of the MBI-C across diverse populations is required. The MBI-C is freely available and can be downloaded in several languages from its official website [https://mbitest.org/lang2.html (accessed on 15 December 2024)], including French, Italian, Spanish, German, Japanese, and Mandarin.

Europe is projected to become the most aged region in the next decades, with adults aged 65 and over expected to comprise one-third of Greece’s population by 2100 [15]. Despite this demographic shift, studies on MBI and MBI-C in Greece are lacking. Cultural factors, such as strong family involvement in older adult care and potential under-reporting of behavioral symptoms due to stigma, may influence symptom recognition and assessment. Moreover, factors such as the Mediterranean diet, physical activity, and social engagement may affect the relationship between MBI and cognitive decline, highlighting the need for investigating MBI in the Greek context. In addition, many regions in Greece are remote or underserved, with limited access to advanced diagnostic tools such as amyloid positron emission tomography (PET) scans [16]. This underscores the need for easy-to-administer clinical screening instruments to identify individuals at risk for cognitive decline in the Greek population.

In this study, we aimed to assess the psychometric properties of the Greek-translated version of the MBI-C, and its ability to differentiate older adults with normal cognition from those with MCI due to AD (MCI-AD) in a memory clinic setting. We hypothesized that the Greek version of the MBI-C would demonstrate satisfactory psychometric properties, except for a few items potentially influenced by cultural factors. In particular, stigma surrounding mental health could lead to the under-reporting of psychotic symptoms such as hallucinations and delusional thoughts, while strong family bonds may hinder the recognition of apathy, as such changes might be misattributed to aging or personality traits.

## 2. Materials and Methods

### 2.1. Study Participants and Clinical Assessment Procedures

A total of 181 older adults aged over 50 years, including 77 healthy participants (HPs) and 104 patients with MCI-AD, accompanied by a close informant, were recruited from the Third Age Day Care Center IASIS during 2019–2023. All participants underwent a clinical interview, detailed neuropsychological assessment, neuroimaging, and other diagnostic work-ups when needed for diagnostic purposes. Close informants, defined as family members, other relatives, caregivers or individuals with regular contact with the participant (at least once a week), completed the MBI-C for each participant. Participants were excluded if they declined to participate, had a diagnosis of dementia, lacked a close informant, were non-Greek speakers, or had a non-AD cause of MCI, as determined by the clinical or diagnostic work-up [e.g., extensive microvascular burden in the brain magnetic resonance imaging (MRI) suggesting vascular cognitive impairment and not MCI-AD]. The raters of the MBI-C were blinded to the MMSE, ACE-R scores, and MCI-AD diagnosis to ensure unbiased assessment.

This study was approved by the Scientific Committee of the Third Age Day Care Center IASIS (date of approval: 20 December 2019, protocol number: 03), and informed consent was obtained from all participants.

### 2.2. Neuropsychological Assessment and Diagnosis of MCI Due to AD

All participants (MCI-AD and HPs) underwent a comprehensive neuropsychological assessment with Mini-Mental State Examination (MMSE) and Addenbrook’s Cognitive Examination-Revised (ACE-R). The Mini-Mental State Examination (MMSE) is the most widely used cognitive screening tool, providing an overall measure of cognitive performance. It consists of 30 items assessing orientation, attention, memory (immediate and delayed recall), language, calculation, and visuoconstructional abilities [17]. The total score ranges from 0 to 30, with lower scores indicating greater cognitive impairment [17]. The ACE-R is a more comprehensive instrument including five subscales for evaluating attention/orientation, memory, verbal fluency, language, and visuospatial abilities [18]. It provides a total score of 100, with higher scores reflecting better cognitive performance [18].

The diagnosis of MCI-AD was based on the 2011 clinical criteria of the NIA-AA [19]. The diagnostic classification (MCI-AD vs. normal cognition) was determined by senior memory specialists at the memory clinic.

### 2.3. Translation of MBI-C into Greek

The MBI-C is the first tool specifically designed to assess MBI in functionally independent older adults, based on the ISTAART-AA criteria [10]. It is a 2-page questionnaire consisting of 34 items, corresponding to the five MBI domains (decreased motivation, emotional dysregulation, impulse dyscontrol, social inappropriateness, and abnormal perception or thought content) [10]. The apathy domain includes 6 items, evaluating cognitive, emotional, and behavioral apathy. The affect domain consists of 6 questions, including 4 items for depressive features of anhedonia, low mood, guilt, and hopelessness, and 1 item each for panic and worry. The impulse dyscontrol domain contains 12 items assessing aggression, agitation, impulsivity, abnormal reward and reinforcement, and recklessness. The social appropriateness domain includes 5 items evaluating sensitivity, tact, and empathy. The abnormal thought and perception domain contains 5 items describing grandiosity, suspiciousness, and auditory and visual hallucinations [10]. The MBI-C can be completed by a close informant (primarily), the patient, or a clinician [10]. Each item requires a “yes” or “no” response, followed by a severity rating (0 = “no”, 1 = mild, 2 = moderate, 3 = severe) [10]. A “yes” response is selected only if the behavior represents an impactful change from the individual’s long-standing usual behavior and has persisted for at least six months, either continuously or intermittently [10]. The total MBI-C score is calculated by summing the scores of all 34 items, and it ranges from 0 to 102. Domain scores are derived by summing the item scores within each respective domain. Higher scores indicate greater behavioral impairment. The MBI-C can be used in both clinical and research settings to detect and monitor emerging NPS in populations pre-dementia, potentially identifying individuals at increased risk for cognitive decline [10]. The MBI-C was developed to provide both a total score and individual domain scores, facilitating future validation and prognostic applications [10]. The MBI-C has been validated in populations with MCI [11] and SCD [12], with cutoff scores of 6.5 and 8.5 points, respectively, for defining MBI. Additionally, its five-factor structure has been validated in older individuals who are cognitively unimpaired, while cutoff scores for this population remain uncertain [13]. The cutoff scores for its separate domains have not been validated yet. The MBI-C is freely available at https://mbitest.org/ (accessed on 15 December 2024).

The translation process followed a structured methodology to ensure linguistic and conceptual equivalence between the English and Greek versions. Two bilingual dementia experts familiar with MBI (J.P., psychiatrist and M.H., clinical neuropsychologist) independently translated the MBI-C from English to Greek. These Greek versions were then back-translated into English by two additional bilingual healthcare professionals experienced in dementia (E.A., neurologist; E.S., neuropsychologist). To ensure conceptual equivalence, the back-translated versions were compared with the original MBI-C to detect and resolve any inconsistencies. Necessary linguistic adjustments were made to ensure that all items were culturally appropriate while preserving their intended meaning. A single Greek version of the MBI-C was then developed. For cognitive debriefing, pilot testing was performed with 10 Greek-speaking individuals without dementia (aged 50 years and above) with varied educational backgrounds. Feedback was collected to evaluate clarity, comprehensibility, and cultural relevance. The Greek version was confirmed to be appropriate and well understood.

### 2.4. Evaluation of Reliability, Validity, and Diagnostic Accuracy of the Greek Version of the MBI-C to Differentiate MCI-AD from NC

The reliability of the Greek version of the MBI-C was assessed through internal consistency, evaluating how well the items correlated both as an overall scale and within each of the five individual MBI domains.

As the MBI-C is the first tool specifically developed to assess MBI, direct comparisons with other instruments could not be clearly made, and no alternative NPS tool was used in our pre-dementia sample. To examine whether the Greek version of the MBI-C accurately measures the indented theoretical construct (MBI) and whether all items are clear and relevant for the Greek population (content validity), an expert review was conducted. A panel of specialists fluent in both Greek (native speakers) and English, with experience in assessing individuals with dementia and pre-dementia states—including a behavioral neurologist (S.P.) and a neuropsychologist (A.D.)—evaluated the scale. A consensus meeting was held to finalize and confirm the conceptual accuracy and appropriateness of the Greek version of the MBI-C, before its delivery to study participants.

To assess the ability of the tool to distinguish between groups (patients with MCI-AD and HPs), which it should be able to differentiate based on the existing literature evidence [20] (known-group validity), the total and domain MBI-C scores were compared between patients with MCI-AD and HPs. Additionally, correlation analyses between the MBI-C total score and cognitive performance were also conducted for the whole group, as well as for subgroups. Sensitivity and specificity analyses were performed to evaluate the discriminative ability of the Greek MBI-C and determine the optimal cutoff point by maximizing the Youden index (sensitivity + specificity − 1).

### 2.5. Statistical Analysis

Demographic and clinical characteristics were analyzed using descriptive statistics, including means and standard deviations for continuous variables as well as frequencies for categorical variables. The Kolmogorov–Smirnov test was used to assess the normality of the distribution of the continuous variables. Comparisons of continuous variables between patients with MCI-AD and HPs were performed using independent samples t-test or the Mann–Whitney test, depending on data normality. The chi-square test was used to explore potential sex differences between groups. Correlations were examined with Pearson’s r or Spearman’s rho, according to the normality of the data. The internal consistency of the MBI-C was evaluated using Cronbach’s alpha to assess its reliability. Receiver operating characteristic (ROC) analysis was performed to examine the scale’s ability to discriminate between patients with MCI-AD and HPs and to identify the optimal cutoff point. Based on this cutoff point, the frequency of MBI was estimated in the total sample and within subgroups stratified by cognitive status (MCI-AD vs. HPs) and sex (women vs. men).

Since age and education differed significantly between the MCI-AD and HP groups, we performed a supplementary binary logistic regression analysis to account for potential confounders. In this model, MBI diagnosis was the dependent variable, MCI-AD status (vs. HPs) was the independent variable, and covariates included age (in years), education (in years), and sex (female vs. male).

The statistical significance level was set at *p* ≤ 0.05. To adjust for the potential inflation of Type I error, Bonferroni correction was applied to account for multiple comparisons between the MCI-AD and HPs groups, as some variables (age, sex, education, disease duration) were not directly related to preplanned hypotheses [21]. Analyses were conducted using IBM Corp. IBM SPSS Statistics for Windows, Version 28.0. Armonk, NY, USA: IBM Corp; 2021.

## 3. Results

### 3.1. Demographics and Clinical Characteristics of Study Participants

A total of 181 participant–informant dyads were recruited, including 104 patients with MCI-AD and 77 HPs, all of whom completed the Greek version of the MBI-C. Demographic characteristics, including age (years), education (years), disease duration (time from symptom onset to assessment, in months), sex (male, female), and global cognitive measures (MMSE and ACE-R) are summarized in Table 1.

HPs were significantly younger and had more years of education compared to patients with MCI-AD (*p* < 0.001). Patients with MCI-AD showed significantly lower scores on the MMSE and ACE-R compared to HPs. After applying Bonferroni correction for multiple comparisons, the difference in ACE-R scores was no longer statistically significant. No significant sex differences were found between the two groups (*p* = 0.878).

### 3.2. The Final Greek Version of the MBI-C

The final Greek version of the MBI-C is presented in Appendix A (Table A1).

### 3.3. Reliability, Validity, and Diagnostic Accuracy of the Greek Version of the MBI-C to Differentiate MCI-AD from HPs

Regarding reliability testing, Cronbach’s α for the overall MBI-C scale was 0.899, indicating excellent internal consistency. Among the five MBI-C domains, decreased motivation demonstrated low internal consistency (α = 0.564), while abnormal perception or thought content showed borderline internal consistency (α = 0.617). Εmotional dysregulation (α = 0.797) and social inappropriateness (α = 0.764) showed acceptable internal consistency, while impulse dyscontrol exhibited excellent internal consistency (α = 0.901).

Regarding content validity, during the consensus meeting, the experts reviewed the Greek version of the MBI-C and confirmed that it accurately measured the indented theoretical construct (MBI) and that all items were clear and relevant for the Greek population.

The total and domain-specific MBI-C scores for HPs and patients with MCI-AD are presented in Table 2. Among the patients with MCI-AD, the highest domain score was observed in impulse dyscontrol, followed by emotional dysregulation, decreased motivation, social inappropriateness, and abnormal perception or thought content. In HPs, the highest scores were noted in emotional dysregulation, followed by impulse dyscontrol, decreased motivation, abnormal perception or thought content, and social inappropriateness. Patients with MCI-AD had significantly higher total MBI-C scores than HPs [median (Mdn) = 12.5, interquartile range (IQR) = 12 vs. 3 ± 4, respectively, *p* < 0.001). In addition, patients with MCI-AD scored significantly higher across all five MBI-C domains compared to HPs [MCI-AD vs. HPs, emotional dysregulation: 4.5 ± 5 vs. 2 ± 2; impulse dyscontrol: 4 ± 5 vs. 1 ± 2; decreased motivation: 2 ± 5 vs. 0 ± 0; social inappropriateness: 0 ± 2 vs. 0 ± 0; abnormal perception or thought content: 0 ± 1 vs. 0 ± 0, all *p* < 0.001). These differences remained statistically significant after Bonferroni correction for multiple comparisons.

Correlation analysis using Spearman’s rho revealed a significant negative correlation between total MBI-C score and MMSE (ρ = −0.430, *p* < 0.001), as well as between total MBI-C score and ACE-R (ρ = −0.532, *p* < 0.001) for the whole sample. In the HP group, correlation analysis did not reveal a significant association between the total MBI-C score and MMSE (ρ = 0.061, *p* = 0.601) or between the total MBI-C score and ACE-R (ρ = 0.084, *p* = 0.475). Similarly, in participants with MCI-AD, no significant correlation was found between the total MBI-C score and MMSE (ρ = 0.054, *p* = 0.585),or between the total MBI-C score and ACE-R (ρ = −0.012, *p* = 0.903).

To assess the ability of the Greek version of the MBI-C to discriminate between patients with MBI-C and HPs, ROC analysis was performed. The area under the curve (AUC) was 0.871 [SE = 0.026, 95% CI (0.819, 0.923), *p* = < 0.001], indicating excellent diagnostic accuracy. This suggested that the likelihood of correctly classifying a random pair consisting of one individual with MCI-AD and one healthy participant was 87.1%. The optimal cutoff point, determined by maximizing the Youden Index, was 9.5, yielding a sensitivity of 64.4% and a specificity of 97.3%. This cutoff provides a balance between identifying true positives and minimizing false positives. Additional cutoff values were explored to offer alternative trade-offs: a cutoff score of 8.5 demonstrated 74.0% sensitivity and 89.3% specificity, while a lower cutoff of 7.5 increased sensitivity to 77.9%, but reduced specificity to 82.7% (Table 3).

**Figure 1:**
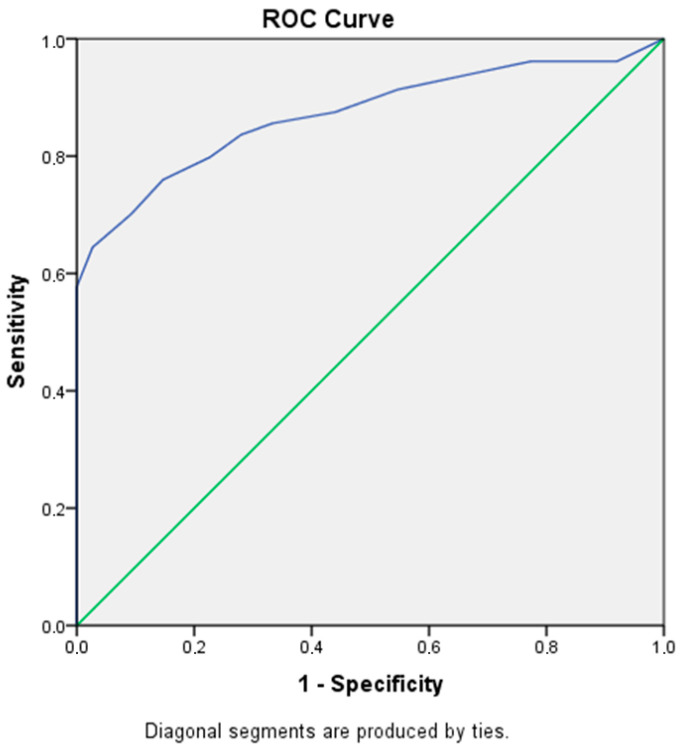
Receiver operating characteristic (ROC) curve illustrating the sensitivity and 1–specificity of the Mild Behavioral Impairment Checklist (MBI-C) in distinguishing cognitively normal participants from those with mild cognitive impairment due to Alzheimer’s disease (MCI-AD). The diagonal dashed line represents random prediction (AUC = 0.5).

Figure 1 represents the ROC curve illustrating the discriminating performance of the MBI-C by plotting sensitivity (true positive rate) against 1–specificity (false positive rate) across all possible cutoff points. The AUC reflects excellent overall accuracy.

### 3.4. MBI Frequency and Participant Characteristics by MBI Status

Applying the cutoff point of 9.5, we calculated the frequency of MBI in the overall sample as well as in the subgroups stratified by cognitive status (MCI-AD vs. HPs) and sex (female vs. male) (Table 4). MBI was present in 40.2% of the total sample, with a frequency of 67.3% in the MCI-AD group and 2.7% in the HP group. When stratified by sex, MBI was observed in 37.3% of women and 44.9% of men. The demographic and clinical characteristics of participants, based on MBI status, are also presented in Table 4. MBI was more frequent in the MCI-AD group than in the HPs (*p* < 0.001). No significant difference in MBI frequency was found between women and men (*p* = 0.35). Participants with MBI were significantly older, had lower education levels, and demonstrated poorer cognitive performance on the MMSE and ACE-R (*p* < 0.001).

In the binary logistic regression analysis, where MBI diagnosis was defined as a total MBI-C score of ≥9.5, the association between MCI-AD and MBI remained statistically significant after adjusting for sex, age, and education (OR: 58.39, 95% CI: 12.91–264.06, *p* < 0.001).

## 4. Discussion

In this study, we demonstrated that the Greek version of the MBI-C, as a whole scale, is valid, with excellent reliability and ability to distinguish between patients with MCI-AD and older adults without cognitive impairment. The strong psychometric properties support its appropriate use in the older Greek population pre-dementia, particularly among patients with MCI-AD, for early detection and timely intervention in the preclinical and prodromal stages of AD.

Internal consistency analysis showed excellent reliability for the Greek version of the overall MBI-C (Cronbach’s α = 0.899). Similar studies in Taiwan [22] (α = 0.893) and Turkey [23] (α = 0.810) also reported good internal consistency for the MBI-C in patients with MCI and cognitively normal individuals. Another study in patients with MCI and mild AD found good reliability (α = 0.895) [24], and the Chinese version of the MBI-C also showed strong internal consistency in patients with AD dementia (α = 0.936) [25].

The reliability across the MBI domains varied in our study: impulse dyscontrol exhibited the highest internal consistency (α = 0.901), while decreased motivation and abnormal perception or thought content had lower Cronbach’s α values (α = 0.564 and 0.617, respectively). The strong reliability of impulse dyscontrol suggests that symptoms like aggression and impulsivity might be more easily recognized by informants due to their disruptive nature. Additionally, Greek social norms emphasize self-restraint and appropriate behavior, particularly among older adults, making deviations from these expectations more noticeable to caregivers, thus enhancing reliability.

Abnormal perception or thought content is a heterogeneous domain, encompassing symptoms such as hallucinations, paranoia, and delusional thinking, which may manifest differently across patients. The low number of positive responders in this domain likely contributed to the borderline reliability. Importantly, psychotic-like features are relatively less common in prodromal AD than in other conditions, such as prodromal dementia with Lewy bodies [26]. Hence, the fact that our sample included only patients with MCI-AD and HPs might have also contributed to the relatively low internal consistency in this domain. In addition, in Greece, psychotic-like symptoms are often stigmatized or less openly discussed within families, which may lead to inconsistent reporting.

The low internal consistency for decreased motivation may stem from informants’ difficulty in recognizing subtle signs of apathy, social withdrawal, and loss of interest, which may be misattributed to normal aging or external factors like retirement. In Greek culture, strong family ties often provide emotional and practical support, potentially masking mild manifestations of decreased motivation.

Consistent with our findings, although the overall internal consistency of the MBI-C has been reported as being high across studies, the reliability of the individual domains tends to vary. For example, the Chinese version of the MBI-C demonstrated excellent internal consistency for the total scale (α = 0.936); however, the domain of social inappropriateness showed a comparatively lower reliability (α = 0.664) [25]. This lower value may be partly attributed to the limited social engagement of older adults in China, which could make it more challenging for informants to observe and report socially inappropriate behaviors [25]. Analogously, both Greek and Chinese cultures are characterized by strong intergenerational support systems and a deep-rooted sense of duty to care for older family members. While this familial closeness can enhance caregiving, it may also obscure the recognition of subtle behavioral changes, such as decreased motivation or social withdrawal, especially when these changes are interpreted as expected consequences of natural aging or loss of routine. Additionally, perceptions of mental illness differ between the two cultures. In China, mental illness has long been a taboo subject, though public health efforts have recently attempted to reduce stigma [27]. In Greece, while awareness has increased, there remains a tendency to attribute behavioral disturbances to external life stressors or temperament, which may lead to variability in how informants interpret and report NPSs. Such cultural differences likely contribute to the variability in internal consistency across MBI-C domains observed in different versions of the tool, highlighting the importance of culturally adapted instruments and interpretive frameworks when assessing NPSs in populations with pre-dementia.

Collectively, these findings suggest that while the Greek MBI-C is a robust tool overall, certain domains may benefit from additional measurement approaches, cultural adaptations, or informant training to better identify NPSs in those with MBI. Future studies should explore ways to enhance the internal consistency in these domains, involving qualitative methodology via interviews to deeper understand the sociocultural factors, adapt the relevant items, and subsequently optimize the cultural adaptability of the tool. In addition, further research in multi-cause MCI is required to clarify the reliability of these domains.

Health issues, including mental health, are perceived and expressed differently across cultures [28]. For example, some cultures may suppress emotional expression to avoid intensifying distress, while others, like many Asian cultures, may refrain from seeking help due to shame or family honor concerns [29]. Additionally, cultural factors shape the presentation of NPSs; for example, obsessive-compulsive behaviors vary by region, with aggressive behaviors more common in Brazil and religious obsessions prevalent in the Middle East [30]. Given that MBI involves recent and often mild behavioral changes, incorporating sociocultural factors into its assessment is crucial for accurately identifying and interpreting NPSs across diverse populations.

In agreement with the previous literature, total and domain MBI-C scores were higher in patients with MCI-AD compared to HPs, and total MBI-C scores were negatively correlated with both MMSE and ACE-R in the whole sample [25,31], supporting known-group validity. The lack of statistically significant correlations between MBI-C scores and cognitive performance within each subgroup (patients with MCI-AD, HPs), when analyzed separately, may be attributed to the limited variability or smaller sample sizes within the individual groups. Among the different MBI domains, emotional dysregulation and impulse dyscontrol received the highest scores in the HP and MCI-AD groups, respectively. These findings are consistent with the broader literature on MBI, with a recent meta-analysis indicating that emotional dysregulation, followed by impulse dyscontrol, is the most commonly affected MBI domain in both MCI and cognitively normal groups. Decreased motivation, social inappropriateness, and psychotic features were the least prevalent MBI domains in both groups, which also agrees with the relevant literature [32]. Impulse dyscontrol, which includes symptoms such as irritability, aggression, reckless driving, frequent interruptions, gambling, and other risky behaviors, may be more pronounced and concerning in individuals with concurrent cognitive decline, potentially making it more likely to be reported by informants. The mean time from symptom onset to the first medical assessment for patients with MCI and mild AD dementia has been previously reported as approximately 15 months [33]. In our study, the mean disease duration for MCI-AD was 30.12 ± 19.10 months, suggesting a more prolonged disease course, during which impulse control symptoms may become more evident compared to earlier, subtler emotional changes. Additionally, older Greek adults may be less likely to seek early medical intervention for mild cognitive issues compared to populations in English-speaking countries, where most MBI studies have been conducted, which could help explain our findings.

The Greek MBI-C demonstrated excellent diagnostic accuracy, with an AUC of 0.871, effectively distinguishing patients with MCI-AD from individuals with normal cognition. The optimal cutoff score of 9.5 yielded a sensitivity of 64.4% and specificity of 97.3%, indicating a low false-positive rate. A study using the Chinese version of the MBI-C identified an optimal cutoff score of 6/7 for detecting Alzheimer’s disease dementia, yielding a sensitivity of 86.96% and a specificity of 86.00% [25]. The discrepancy in cutoff scores, sensitivity, and specificity between that study and ours may be attributed to differences in the study populations: while the Chinese study included patients with AD dementia across a range of severities (mild to severe), our sample consisted of individuals with MCI due to AD, representing an earlier disease stage.

In our study, alternative cutoffs were 7.5 (sensitivity 77.9%, specificity 82.7%) and 8.5 (sensitivity 74%, specificity 89.3%). The 9.5 cutoff is ideal for minimizing false positives. However, for screening and early detection, prioritizing sensitivity (e.g., using a cutoff of 8.5) may be more beneficial to identify more MCI-AD cases, even at the cost of higher false positives. The optimal cutoff should be chosen based on the clinical or research context, in combination with additional tools, including comprehensive neuropsychological assessment, neuroimaging, and/or other biomarkers, since the MBI-C is typically employed to detect behavioral symptoms that may signal an increased risk for cognitive decline, and it cannot be utilized as a standalone diagnostic tool for MCI.

Using the MBI-C total cutoff of 9.5, participants with MBI were older, less educated, had lower cognitive performance, and were more likely to receive an MCI-AD diagnosis. Although MBI was more prevalent in men than women in our study (44.9% vs. 37.3%), the difference was not statistically significant. Given the inconsistent findings in the literature regarding sex differences in MBI prevalence [34], larger, community-based studies are needed to clarify potential sex-related patterns in the Greek population. Importantly, the MBI-C has been validated in populations with MCI [11] and SCD [12], with cutoff scores of 6.5 and 8.5 points, respectively, for defining MBI. Future studies should also define the optimal cutoffs for defining MBI in the Greek population.

Unsurprisingly, patients with MCI-AD were older, less educated, and exhibited lower cognitive performance. The association between MBI and MCI-AD remained significant even after adjusting for age, sex, and education in the logistic regression model, reinforcing the strong relationship between MCI-AD and MBI, independent of these factors. Nevertheless, age and education may still indirectly influence the recognition and reporting of NPSs. Older individuals may be more likely to exhibit behavioral symptoms due to an underlying neurodegenerative disease, while lower educational levels in patients may influence the presentation or expression of such symptoms. Education level could also be linked to cognitive reserve, potentially modifying both symptom severity and the timing of clinical recognition [35]. Age and education should be taken into consideration when interpreting results and designing future studies aiming to refine normative data and cutoff scores for diverse demographic groups.

Our study has several limitations. First, our sample included only HPs and patients with MCI due to AD, limiting generalizability to other MCI causes, such as vascular neurocognitive disorder, Lewy body disease, and frontotemporal lobar degeneration. Cognitive fluctuations, complex visual hallucinations, and systematized delusions such as Capgras syndrome are more likely to occur in prodromal dementia with Lewy bodies compared to in individuals with prodromal AD [26]. Apathy, loss of insight, impaired social cognition, somatization, delusions, and hallucinations are also especially common in the prodromal stages of frontotemporal dementia [36]. These discrepancies might influence symptom presentation, identification, and reporting. Therefore, since partially distinct NPS profiles are observed in different prodromal dementia syndromes, future studies should evaluate the validity, reliability, and applicability of the Greek MBI-C across diverse etiological MCI subtypes in a more heterogeneous clinical population.

In addition, MCI-AD diagnosis was based on the 2011 NIA-AA clinical criteria [19] rather than the updated 2024 NIA-AA research framework, which incorporates biomarkers [8]. While biomarkers like CSF analysis, amyloid and tau PET, and blood-based markers could enhance diagnostic accuracy, they are not widely available, highlighting the continued relevance of clinical diagnosis and the use of MBI-C in real-world practice, particularly in underserved areas. Test–retest and inter-rater reliability could not be assessed, as repeated measurements and multiple raters were not used in our study. Furthermore, the absence of self-filled MBI-C data limited our ability to compare individual vs. informant reports [37], which should be addressed in future research. Additionally, our cross-sectional design prevented us from examining the predictive value of MBI-C for cognitive decline, warranting further investigation in longitudinal studies.

Another limitation is that we did not use any other tool for NPSs to assess MBI, such as NPI or NPI-Q, which would strengthen validity. Although these instruments have often been used for defining MBI in several studies, their applicability in functionally independent populations without dementia is disputed, as they may lack the specificity for detecting early and subtle behavioral or personality changes. The time reference in these instruments is also only one month (versus the six-month period according to the definition of MBI). MBI-C is the first established tool to define and assess MBI, highlighting that comparisons with existing tools cannot be clearly made. However, despite these limitations, it would be useful for future studies to include NPI or NPI-Q in the study design to enhance the clinical credibility of the Greek MBI-C. Nevertheless, the confirmed content validity via expert review and known-group validity, the excellent overall internal consistency and diagnostic accuracy support its applicability in the Greek population with MCI-AD.

Although some participants underwent neuroimaging for diagnostic purposes, these data were not included in our study, as exploring the associations between MBI-C scores and brain atrophy or other neuroimaging markers was not part of our research objectives. Future studies could examine these relationships to further understand the neurobiological underpinnings of MBI and its potential as an early indicator of neurodegenerative diseases in the Greek population. One of the key strengths of our study is that it is the first to examine the validity, reliability and diagnostic accuracy of the Greek translated version of the MBI-C in distinguishing between patients with MCI-AD and individuals with normal cognition. Furthermore, the use of a well-characterized clinical sample and comprehensive cognitive assessments reduces potential diagnostic misclassifications and enhances the reliability of our findings. The inclusion of patients with MCI due to AD minimizes the potential confounders from other MCI subtypes, enhancing the generalizability of our results to this population, which is one of the most common causes of cognitive impairment in aging adults. Moreover, our discussion on Greek social norms and family dynamics provide valuable insights for future research to examine how cultural factors may affect the recognition and reporting of NPSs in the population pre-dementia.

The strong psychometric properties of the Greek version of the MBI-C holds significant clinical relevance. As a brief tool designed to detect emergent and persistent NPSs in later life, it allows for the early identification of individuals at increased risk for cognitive decline. Given its ease of use, it can support neurologists, psychiatrists, and general practitioners in recognizing subtle behavioral changes that often precede measurable cognitive deficits, particularly in patients with MCI-AD or even those with normal cognition. Moreover, the tool can improve diagnostic accuracy and risk stratification in memory clinics, guide patient monitoring over time, and facilitate timely, personalized intervention strategies and care planning. Its application is especially valuable in real-world clinical practice in Greece, including rural and underserved areas, where access to specialized neuropsychiatric evaluation and biomarker testing may be limited [16].

Future research should focus on investigating the psychometric properties of the Greek MBI-C in larger and more diverse samples, including individuals with SCD, and non-AD causes of MCI. In addition, studies in various Greek-speaking populations with different educational levels and from diverse regions will enhance the generalizability of the Greek MBI-C. This can help identify whether demographic or sociocultural factors influence specific MBI domains and refine cutoff scores for different subgroups. Cross-cultural comparisons between the Greek MBI-C and versions in other languages may reveal how cultural factors affect the expression and interpretation of NPSs.

Longitudinal studies are also needed to assess the predictive value of MBI symptoms for future cognitive decline and dementia conversion. Additionally, integrating biomarker data like amyloid and tau PET or blood-based markers could improve the diagnostic utility of the MBI-C for early AD detection. In this context, it is recommended for future studies to verify the pathological association by combining AD biomarkers, such as amyloid PET or plasma phosphorylated tau 217 (pTau-217) [38,39], with a longitudinal study design, such as half-year evaluations, to clarify the predictive value and clinical application potential of the Greek MBI-C in dementia conversion. To improve symptom reporting, culturally tailored MBI assessment guidelines, including training for informants and caregivers, should be developed. Given the subjective nature of informant-based assessments, future studies could explore digital tools using ecological momentary assessment (EMA) to capture real-time, even subtle, behavioral alterations in the individual’s natural environment, reducing recall bias [40]. Artificial intelligence (AI)-driven tools to analyze speech patterns for detecting motivational or emotional changes may be also valuable. Finally, comparing the Greek MBI-C with other NPS tools, such as the NPI or NPI-Q, could refine its role in routine clinical practice and optimize its use in screening aging populations for NPSs.

## 5. Conclusions

In conclusion, the Greek version of the MBI-C, as an overall instrument, is a valid and reliable tool for assessing MBI in the older Greek population with MCI-AD. However, the domains of decreased motivation and abnormal perception or thought content warrant further investigation within the Greek sociocultural context. Future research in Greece should explore the tool’s predictive role in dementia conversion and its psychometric properties in broader populations including SCD and non-AD causes of MCI.

## Figures and Tables

**Table 1:** Demographic and neurocognitive characteristics of study participants.

Characteristic	Healthy Participants (*n* = 77)	Patients with MCI-AD (*n* = 104)	*p*-Value
Age (years), Mdn ± IQR	71 ± 13	77 ± 9	*p* < 0.001 *
Disease duration (months), M ± SD	-	30.12 ± 19.10	
Sex (male/female%)	37.7/62.3	39.4/60.6	*p* = 0.878 ***
Education (years), Mdn ± IQR	14 ± 4	10.44 ± 4.05	*p* < 0.001 *
MMSE, Mdn ± IQR	29 ± 2	26 ± 2	*p* < 0.001 *
ACE-R, M ± SD	92.27 ± 3.40	72.77 ± 8.93	*p* < 0.05 **

* Mann–Whitney U test, ** *t*-test, *** chi-square test. M, mean; SD, standard deviation; Mdn, median; IQR, interquartile range; MCI-AD, mild cognitive impairment due to Alzheimer’s disease; MMSE, Mini-Mental State Examination; ACE-R, Addenbrook’s Cognitive Examination—Revised.

**Table 2:** Total and domain MBI-C scores of study participants.

MBI-C Score	Healthy Participants (*n* = 77)	Patients with MCI-AD (*n* = 104)	*p*-Value
Total, Mdn ± IQR	3 ± 4	12.5 ± 12	*p* < 0.001 *
Emotional Dysregulation, Mdn ± IQR	2 ± 2	4.5 ± 5	*p* < 0.001 *
Impulse Dyscontrol, Mdn ± IQR	1 ± 2	4 ± 5	*p* < 0.001 *
Decreased Motivation, Mdn ± IQR	0 ± 0	2 ± 5	*p* < 0.001 *
Social Inappropriateness, Mdn ± IQR	0 ± 0	0 ± 2	*p* < 0.001 *
Abnormal Perception or Thought Content, Mdn ± IQR	0 ± 0	0 ± 1	*p* < 0.001 *

* Mann–Whitney U test. M, mean; SD, standard deviation; Mdn, median; IQR, interquartile range; MCI-AD, mild cognitive impairment due to Alzheimer’s disease; MBI-C, Mild Behavioral Impairment Checklist (MBI-C).

**Table 3:** Cutoff points for the total score on the Greek version of the Mild Behavioral Impairment Checklist (MBI-C) based on different combinations of sensitivity and specificity in the receiver operating characteristic (ROC) analysis for discriminating participants with mild cognitive impairment due to Alzheimer’s disease (MCI-AD) and healthy participants (HPs).

Cutoffs for Total MBI-C Score	Sensitivity	Specificity
4.50	85.6	66.7
5.50	84.6	72.0
6.50	79.8	74.7
7.50	77.9	82.7
8.50	74.0	89.3
9.50 (Youden index optimal)	67.3	97.3
10.50	60.6	100.0

**Table 4:** Demographic and clinical characteristics of participants based on MBI status.

Group Reference for MBI Frequency/ Characteristic	MBI	No-MBI	*p*-Value
Whole Sample (*n*, %)	72 (40.2%)	107 (59.8%)	
Stratified by sex			*p* = 0.35
Women (*n*, %)	41/110 (37.3%)	69/110 (62.7%)	
Men (*n*, %)	31/69 (44.9%)	38/69 (55.1%)	
Stratified by cognitive status			*p* < 0.001
MCI-AD (*n*, %)	70/104 (67.3%)	34/104 (32.7%)	
HPs (*n*, %)	2/75 (2.7%)	73/75 (97.3%)	
Characteristic			
Age (years), M ± SD	77.72 ± 6.26	71.33 ± 9.36	*p* < 0.001 **
Education (years), M ± SD	10.42 ± 3.95	12 ± 7	*p* = 0.002 *
MMSE, M ± SD	26.28 ± 1.60	28 ± 2	*p* < 0.001 *
ACE-R, M ± SD	72.88 ± 9.87	90 ± 12	*p* < 0.001 *

* Mann–Whitney U test, ** *t*-test. M, mean; SD, standard deviation; Mdn, median, IQR, interquartile range; MCI-AD, mild cognitive impairment due to Alzheimer’s disease; HPs, healthy participants; MBI-C, Mild Behavioral Impairment Checklist (MBI-C); MMSE, Mini-Mental State Examination; Addenbrooke’s Cognitive Examination—Revised, ACE-R.

## Data Availability

The data presented in this study are available on request from the corresponding author due to privacy and ethical reasons.

## Abbreviations

The following abbreviations are used in this manuscript:

MBI	Mild behavioral impairment
AD	Alzheimer’s disease
MBI-C	Mild Behavioral Impairment Checklist
MCI-AD	Mild cognitive impairment due to Alzheimer’s disease
HPs	Healthy participants
MMSE	Mini-Mental State Examination
ACE-R	Addenbrooke’s Cognitive Examination—Revised
ROC	Receiver operating characteristic
AUC	Area under the curve
NPS	Neuropsychiatric symptom
bvFTD	Behavioral variant frontotemporal dementia
SCD	Subjective cognitive decline
NIA-AA	National Institute on Aging and Alzheimer’s Association
ISTAART-AA	International Society to Advance Alzheimer’s Research and Treatment—Alzheimer’s Association
SPSS	Statistical Package for the Social Sciences
SE	Standard error
CI	Confidence interval
SD	Standard deviation
NPI-Q	Neuropsychiatric Inventory Questionnaire
CSF	Cerebrospinal fluid
PET	Positron emission tomography
FDG	Fluorodeoxyglucose
OR	Odds ratio

## Appendix A

**Table A1:** The Greek version of the Mild Behavioral Impairment Checklist (MBI-C).

Ερωτηματολόγιο Ήπιας Συμπεριφορικής Διαταραχής—Mild Behavioral Impairment—Checklist (MBI-C) (ελληνική έκδοση, μετάφραση: Ι. Παπατριανταφύλλου, MD, PhD)				
OΝOΜA: HΜΕΡOΜHΝΙA: Ερευνήσατε αν τους τελευταίους 6 μήνες υπάρχουν τα παρακάτω συμπτώματα και βαθμολογήσατε την σοβαρότητά τους 0 δεν υπάρχει, 1 ήπια, 2 μέτρια, 3 σοβαρή (σε αμφιβολία την σοβαρότερη βαθμολογία)	ΣOΒAΡOΤHΤA			
**AFF** **Aυτό το πεδίο περιγράφει τη διάθεση και τα συμπτώματα άγχους**	*όχι*	*ναι*		
1. Το άτομο έχει αναπτύξει θλίψη ή φαίνεται έχει κακή διάθεση; Έχει επεισόδια που κλαίει/ξεσπά σε κλάματα;	0	1	2	3
(cont.)				
2. Το άτομο δυσκολεύεται/είναι λιγότερο ικανό να αντλήσει ευχαρίστηση;	0	1	2	3
3. Το άτομο είναι αποθαρρημένο για το μέλλον του ή νιώθει αποτυχημένος;	0	1	2	3
4. Το άτομο βλέπει τον εαυτό του ως βάρος για την οικογένειά του;	0	1	2	3
5. Το άτομο είναι περισσότερο αγχωμένο ή ανήσυχο για πράγματα που αποτελούν ρουτίνα (π.χ. εκδηλώσεις, επισκέψεις);	0	1	2	3
6. Το άτομο νιώθει μεγάλη ένταση, αδυνατεί να χαλαρώσει, παρουσιάζει αστάθεια/τρόμο ή έχει συμπτώματα πανικού;	0	1	2	3
**MOT** **Aυτό το πεδίο περιγράφει το ενδιαφέρον, την παρακίνηση και τη δράση/ενέργεια**	*όχι*	*ναι*		
1. Το άτομο έχει χάσει το ενδιαφέρον για τους φίλους, την οικογένεια ή τις δραστηριότητες του σπιτιού;	0	1	2	3
2. Το άτομο έχει χάσει την περιέργεια του για θέματα που συνήθως τραβούσαν το ενδιαφέρον του/της;	0	1	2	3
3. Το άτομο έχει γίνει λιγότερο αυθόρμητο ή ενεργό/ δραστήριο- για παράδειγμα είναι λιγότερο πιθανό να ξεκινήσει ή να διατηρήσει μία συζήτηση;	0	1	2	3
4. Το άτομο έχει χάσει το κίνητρό για τις υποχρεώσεις που είχε μέχρι τώρα και για τα ενδιαφέροντά του;	0	1	2	3
5. Το άτομο είναι λιγότερο στοργικό και/ή χωρίς συναισθηματική ανταπόκριση σε σχέση με παλαιότερα;	0	1	2	3
6. Πλέον δεν νοιάζεται για τίποτα;	0	1	2	3
**IMP** **Aυτό το πεδίο περιγράφει την ικανότητα καθυστέρησης στην λήψη ικανοποίησης, τον έλεγχο της** **συμπεριφοράς, την παρόρμηση, την πρόσληψη τροφής και/ή αλλαγές στην επιβράβευση**	*όχι*	*ναι*		
1. Το άτομο έχει γίνει ανήσυχο, επιθετικό, ευερέθιστο ή κυκλοθυμικό;	0	1	2	3
2. Έχει γίνει αναίτια ή περίεργα/ ιδιαίτερα εριστικό;	0	1	2	3
3. Το άτομο έχει γίνει περισσότερο παρορμητικό, φαίνεται ως εάν να λειτουργεί χωρίς να υπολογίζει τίποτα;	0	1	2	3
4. Το άτομο παρουσιάζει σεξουαλικές απαγορευμένες ή αδιάκριτες συμπεριφορές, όπως το να ακουμπάει (τον εαυτό του ή άλλους), να αγκαλιάζει, θωπεύει κ.α., με τρόπο που δεν είναι στο χαρακτήρα του και μπορεί να προκαλέσει παράπτωμα;	0	1	2	3
5. Το άτομο απογοητεύεται πιο εύκολα ή είναι πιο ανυπόμονο; Έχει δυσκολία να αντιμετωπίσει τις καθυστερήσεις ή να περιμένει σε εκδηλώσεις για τη σειρά του;	0	1	2	3
6. Το άτομο παρουσιάζει μία καινοφανή απερισκεψία ή έλλειψη κρίσης όταν οδηγεί (π.χ. αναπτύσσει ταχύτητα, κάνει απρόβλεπτους ελιγμούς, απότομες αλλαγές στη λωρίδα κ.α.)	0	1	2	3
7. Το άτομο έχει γίνει περισσότερο ισχυρογνώμων και άκαμπτο, π.χ. επιμένει πολύ στον δικό του τρόπο, δεν είναι πρόθυμο δεν είναι ικανό να δει ή να ακούσει άλλες οπτικές/απόψεις;	0	1	2	3
8. Το άτομο συσσωρεύει/συγκεντρώνει αντικείμενα, κάτι που δεν έκανε στο παρελθόν;	0	1	2	3
9. Το άτομο έχει αναπτύξει απλές επαναληπτικές συμπεριφορές ή ψυχαναγκασμούς;	0	1	2	3
10. Το άτομο έχει πρόσφατα αναπτύξει δυσκολία να ελέγξει το κάπνισμά του, το αλκοόλ, τη λήψη ναρκωτικών ουσιών, τον τζόγο, ή ξεκίνησε την κλοπή από καταστήματα;	0	1	2	3
11. Υπάρχει αλλαγή στις διατροφικές συνήθειες του ατόμου (π.χ. υπερφαγία, γεμίζει το στόμα του με φαγητό, επιμένει στο να τρώει μόνο συγκεκριμένα φαγητά ή να τρώει φαγητά με συγκεκριμένη σειρά);	0	1	2	3
12. Το άτομο δεν βρίσκει πλέον το φαγητό νόστιμο ή ως κάτι που το ευχαριστεί; Τρώει λιγότερο;	0	1	2	3
**SOCCOG** **Aυτό το πεδίο περιγράφει τη συμμόρφωση στις κοινωνικές νόρμες και κοινωνική προσαρμογή, ** **διακριτικότητα και ενσυναίσθηση**	*όχι*	*ναι*		
1. Το άτομο ανησυχεί λιγότερο για την επίδραση των λέξεων ή των πράξεων του στους άλλους; Έχει γίνει απαθής στα συναισθήματα των άλλων;	0	1	2	3
2. Το άτομο έχει αρχίσει να μιλάει πιο ανοιχτά για πολύ προσωπικά ή ιδιωτικά του ζητήματα/θέματα που συνήθως δεν συζητούσε δημόσια;	0	1	2	3
3. Το άτομο μιλά με αγενή ή ακατέργαστο τρόπο ή κάνει άσεμνα σεξουαλικού περιεχομένου σχόλια που δεν θα τα ανέφερε στο παρελθόν;	0	1	2	3
4. Το άτομο φαίνεται να μη συμμορφώνεται στους κοινωνικούς κανόνες/ γνώμη κάτι που παλαιότερα ακολουθούσε στον δημόσιο ή ιδιωτικό χώρο;	0	1	2	3
5. Το άτομο μιλάει σε ξένους ως εάν να του είναι γνωστοί, ή παρεμβαίνει στις δραστηριότητές τους;	0	1	2	3
**PSY** **Aυτό το πεδίο περιγράφει βαθιά ριζωμένες πεποιθήσεις και αισθητηριακές εμπειρίες**	*όχι*	*ναι*		
1. Το άτομο αναφέρει ότι ακούει φωνές ή μιλά σε φανταστικούς ανθρώπους ή «πνεύματα»;	0	1	2	3
2. Το άτομο αναφέρει ή παραπονιέται για/ή ενεργεί σαν να βλέπει πράγματα (π.χ. ανθρώπους, ζώα ή έντομα) που δεν βρίσκονται στο χώρο π.χ. που είναι φανταστικά/μη υπαρκτά για τους άλλους;	0	1	2	3
3. Το άτομο έχει αναπτύξει πεποιθήσεις ότι βρίσκεται σε κίνδυνο, ή ότι οι άλλοι σχεδιάζουν να του/της κάνουν κακό ή να κλέψουν τα αντικείμενά τους;	0	1	2	3
4. Το άτομο έχει γίνει καχύποπτο για τις προθέσεις και τα κίνητρα των άλλων ανθρώπων;	0	1	2	3
5. Το άτομο έχει μη ρεαλιστικές πεποιθήσεις για τις δυνάμεις του, την υγεία του ή τις ικανότητες του;	0	1	2	3
**ΣΥΝOΛO**				
